# The Relationship between Social Capital in Hospitals and Physician Job Satisfaction

**DOI:** 10.1186/1472-6963-9-81

**Published:** 2009-05-16

**Authors:** Oliver Ommen, Elke Driller, Thorsten Köhler, Christoph Kowalski, Nicole Ernstmann, Melanie Neumann, Petra Steffen, Holger Pfaff

**Affiliations:** 1 Center for Health Services Research Cologne, Faculty of Medicine, University of Cologne, Eupener Str 129, 50933 Cologne, Germany; 2 Department of Medical Sociology, Institute and Polyclinic for Occupational and Social Medicine, Faculty of Medicine, University of Cologne, Eupener Str 129, 50933 Cologne, Germany

## Abstract

**Background:**

Job satisfaction in the hospital is an important predictor for many significant management ratios. Acceptance in professional life or high workload are known as important predictors for job satisfaction. The influence of social capital in hospitals on job satisfaction within the health care system, however, remains to be determined. Thus, this article aimed at analysing the relationship between overall job satisfaction of physicians and social capital in hospitals.

**Methods:**

The results of this study are based upon questionnaires sent by mail to 454 physicians working in the field of patient care in 4 different German hospitals in 2002. 277 clinicians responded to the poll, for a response rate of 61%. Analysis was performed using three linear regression models with physician overall job satisfaction as the dependent variable and age, gender, professional experience, workload, and social capital as independent variables.

**Results:**

The first regression model explained nearly 9% of the variance of job satisfaction. Whereas job satisfaction increased slightly with age, gender and professional experience were not identified as significant factors to explain the variance. Setting up a second model with the addition of subjectively-perceived workload to the analysis, the explained variance increased to 18% and job satisfaction decreased significantly with increasing workload. The third model including social capital in hospital explained 36% of the variance with social capital, professional experience and workload as significant factors.

**Conclusion:**

This analysis demonstrated that the social capital of an organisation, in addition to professional experience and workload, represents a significant predictor of overall job satisfaction of physicians working in the field of patient care. Trust, mutual understanding, shared aims, and ethical values are qualities of social capital that unify members of social networks and communities and enable them to act cooperatively.

## Background

National and international studies in recent years have revealed that a significant number of physicians working in the field of patient care are not satisfied with their job and the associated working conditions [1-3]. A survey conducted in the US, for example, demonstrated this dissatisfaction in revealing that up to 40% of the physicians practicing in hospitals would not take up this profession again. Even a higher portion of the questioned physicians stated that they prevented their children from becoming a physician [4]. The workload of physicians proves to be one of the causes for the situation described. This fact is shown by different North American and European studies [5-10]. Also, the working conditions of clinicians in Germany have changed significantly in recent years. The number of patients in German hospitals has increased from approximately 14 million in 1990 to approximately 17 million in 2004. During the same period, however, the average length of stay has fallen from 14.7 days to 8.7 days [11]. In addition to this development, increasing bureaucracy and mechanisation in daily clinical life, particularly the growing demands relating to documentation and quality control, play a significant role in a physician's practice and result in distancing from the patient [12]. This is not only the case in Germany, but can be seen as an international trend [4,8,13,14]. However, in addition to workload, other factors exist. It is reported that growing patients' needs and economic, organisational, and regulatory factors affect job satisfaction decisively [15]. In particular, many physicians feel their autonomy or their self-conception as physicians are restricted by these factors [16,17]. There exist studies indicating that increasing deprofessionalisation and restriction of professional autonomy evoke job dissatisfaction in physicians [1,16,18-20]. In addition, advanced vocational training facilities (offered on the job) have to be considered [15]. Finally, a linkage exists between a physician's salary and satisfaction with the job [5]. The heavy objective and subjective burdens and the dissatisfaction in practising within the medical profession causes a growing number of physicians, especially young physicians, to change their vocational field [21]. Many studies have demonstrated a link between job satisfaction of physicians and the probability of quitting their job or the frequency of job changes [22-26]. A growing number of physicians try to avert the possible consequences of the workload felt to be too heavy and the dissatisfaction involved by taking up jobs in non-medical professions at an early stage. In particular, stress and burnout are common consequences of regular overwork, which is not merely due to the pure quantity of work, but also due to the quality to be delivered. Being responsible for appropriate and successful therapy and catering to the demands of different social circles (colleagues, relatives, and health insurance) frequently leads to chronic stress, burnout, and physical and other mental diseases [7,27]. Furthermore, a clear relationship between stress, burnout, and job satisfaction could be shown [17,28,29]. This relationship certainly has to be considered as reciprocal: stress and burnout diminish job satisfaction, low job satisfaction in turn intensifies the symptoms of stress and burnout. Obviously, it appears that high job satisfaction, however, can also act as a protective factor and prevent chronic job stress [7,17]. The particular relevance of job satisfaction is demonstrated further by several studies showing links between job satisfaction of physicians and the quality of medical care [23,30-34]. Thus, for example, in addition to the impact of physician job satisfaction on patient satisfaction [23,30] and adherence [23,33], links between the occurrence of errors in treatment and job satisfaction have been described [15]. Although physician job satisfaction has been assessed to a large extent in recent years, very little is known about the effects of organizational characteristics, such as a culture of value and trust, which are expressions of social capital, on overall job satisfaction.

### General definitions of Job Satisfaction and Social capital in the workplace

#### Job satisfaction

Job satisfaction is defined as a global attitude that individuals have towards their jobs [35]. It is an extent to which one feels positively or negatively about different facets of the job e.g. job conditions, co-workers and working time [36-39] and is a complex set of interrelationships of tasks, roles, responsibilities, interactions, incentives and rewards [40].

#### Social capital in the workplace

Social capital can be regarded as a resource which helps people and organizations cope with stress and helps foster salutogenic potential. There are two forms of social capital: 1) individual social capital and collective social capital. An individualistic version of social capital has been defined by Bourdieu [41]. In brief, social capital, according to Bourdieu, is the "aggregate of the actual or potential resources which are linked to possession of a durable network of more or less institutionalized relationships of mutual acquaintance and recognition – or in other words, to membership in a group – which provides each of its members with the backing of the collectively owned capital, a credential which entitles them to credit, in the various senses of the word" [42]. Social epidemiologic research during the last 20 years shows that social relationships, which are experienced as being helpful and positive, promote general well-being and protect against physical harm [43,44]. Coleman [45] described the collective version of the term "social capital" as follows: "Unlike other forms of capital, social capital inheres in the structure of relations between persons and among persons. It is lodged neither in individuals nor in physical implements of production." Given this definition, it can be assumed that not only individuals, but also complex organizations, such as hospitals, possess social capital. Its components are, in particular, the existence of collective values and convictions and mutual trust between the members of an organization [46]. Collective social capital can be defined as a feature of social systems that is able to improve the health and the capacity to perform of its members [47]. Research into support and networks has also shown that a person's social network has a significant impact on his or her performance capacity, health, and emotional balance. The stability, scope, and functionality of social networks have a modulating effect on cognition, motivation, and emotions [48-53]. A successfully established atmosphere of trust and a feeling of common values and convictions may help people work together and make it easier for them to assess the conditions of their daily work by reducing insecurity, uncertainty, and disorientation, and to improve their performance. Social capital is generated from internalized, informal standards within an organization and produces cooperation [54-57]. Putnam and Coleman regard social capital as a way of solving collective problems through a sense of community and trust. The inherent potential for people to exploit others in the production of collective goods is reduced by activity structures that are governed by reciprocity standards [55].

### Aim of the study

Little empirical work has been published using the concept of social capital in the health care industry [58]. However, the studies which have been published on this topic yet refer to the particular importance of social capital in the health care sector, eg. in the inpatient or ambulatory sector. Thus, Waisel demonstrates in his study how social capital improves the operating room working environment and therefore increases efficiency and quality of patient care [59]. DiCicco et al [60] developed a model of social capital to enhance relationships within primary care practices that promote organizational success and improve patient care outcomes. Hoelscher, Hoffman & Dawley [61] reviewed the literature and showed that existing social capital leads to competitive advantage and enhanced medical group performance. Hofmeyer & Marck [62] outlined the role of social capital for organizational integrity, healthy workplace cultures, sustainable resource management, improved nurse retention, effective knowledge translation, and safer patient care. Research into the relationship between social capital in hospitals and job satisfaction of clinicians is however still at an early stage. To our knowledge, there is no existing literature to date that has explicitly examined the relationship between social capital in hospitals and physician job satisfaction. Therefore, the aim of the current study was to examine the effects of collective social capital at the workplace on overall job satisfaction of clinicians. We hypothesized that a significant relationship exists between social capital in the hospital and physician satisfaction, assuming that this relationship can be proved not only in bivariate form, but persists after controlling for socio-demographic factors, such as age, gender, professional experience, and subjective workload.

## Methods

The following analysis is based on data from a project entitled "Corporate Governance Using Biopsychosocial Indicators" (CoBI) study, funded by the German Federal Ministry of Education and Research. The "Biopsychosocial Indicators for Employees Questionnaire" (BIQ) used herein was especially developed for this study. It contains both internationally-established instruments, such as the "Maslach Burnout Inventory," and scales especially developed for this and further studies, among them the social capital scale described below. It consists of valid indicators of how employees regard their work situation and their organization [63].

### Ethics

This study was approved by the Research Ethics Board at the University of Cologne. All participants provided informed consent for the survey.

### Participants and procedure

The CoBI study surveyed clinicians, nursing staff, and administrative and technical employees. In 2002 a total of 2,644 employees representing four German hospitals received an anonymous questionnaire by post. These employees had been working full-time or part-time in one of the four hospitals during the survey period. Further inclusion and exclusion criteria were not defined. Two hospitals in East Germany and two hospitals in West Germany, two of which offer maximum healthcare services and two of which offer basic healthcare services, were included (Table 1). Of the 2,644 employees, 454 were clinicians. 277 clinicians responded to the poll, for a response rate of 61%. This population made up the sample for the present study.

**Table 1:** Structures of the selected hospitals

	**Hospital 1**	**Hospital 2**	**Hospital 3**	**Hospital 4**
**Region**	West	West	East	East
**Care level**	Max. care	Basic care	Max. care	Basic care
**Specialist departments**	23	6	25	7
**Beds**	1.500	353	1.855	454
**Number of cases in 2001**	47.673	9.437	58.841	15.089

### Measures

#### Overall Job Satisfaction

Following Scarpello and Campell [64], Wanous et al. [65] and Nagy [66] we decided to measure overall job satisfaction taking a single item approach. The most frequently argued advantages of single item measures in contrast to multi-item, multi-dimensional instruments measuring overall job satisfaction are the following: single item measures are much shorter and take up less space, are more cost-effective, may contain more face validity, appear to be correlated fairly with multi-item measures of overall satisfaction and may be better to measure changes in job satisfaction. Furthermore, the problem to operationalize job satisfaction – similar to patient satisfaction – is to integrate all factors influencing job satisfaction in one comprehensive instrument according to their individual weighting. In particular due to the lack of knowledge of the completeness of all potential influence factors and the lack of empirical and theoretical information about their individual weighting, a single item approach seems to be the more appropriate method. Highhouse and Becker [67] e.g. found that facets such as company loyalty, enjoyment of work, and job significance were not captured by a composite facet measure, but were considered in making a global judgment about job satisfaction. Therefore the used variable, "job satisfaction" [68], is based on a homonymous item worded as follows: "If you consider everything that matters in your job (e.g., kind of work, working conditions, colleagues, and working time), how satisfied are you with your job?" Subjective complaints were assessed with a seven-point Likert-type scale with smiley/sad faces above each point.

#### Sociodemographic characteristics and workload

Information about age, gender, and years of professional experience was provided by the respondents. The variable, "workload," based on the scale, "intensity of labour" according to Richter et al. [69], was designed to measure the workload of physicians (Cronbach's α: .78). The six items of the scale were worded as follows: 1) "The required pace of work is very fast," 2) "The tasks are often very difficult to cope with," 3) "I often have very much work to be done," 4) "Usually time is too short and I often work under time pressure," 5) "I'm often exposed to physical strain," and 6) "Too much work has to be done at the same time." Answers ranged from "disagree" to "agree," with each response category measured by a score from 1–4 points; all item values were summed and divided by the number of items.

#### Social capital in hospitals

The variable, "social capital in hospitals," was designed to measure two key features of social capital: 1) common values and 2) perceived trust at the hospital [64]. We used six items to measure this variable [68], e.g., "Agreement and consent dominate in our hospital" and "At our hospital we trust each other." The items were developed using basic sociological principles and central statements relating to social capital described by Coleman [45,54], Putnam [55], and Fukuyama [56]. Respondents could choose from four given responses and each response was assigned points ranging from 1–4; the total scores ranged from 6–24 points. The central determinants of this variable were divided into three types: common values, perceived trust, and reciprocity. Agreement and consent (item 1) and the presence of a "sense of unity" (item 3) represent a common value base [57]. One of the prerequisites for cooperative action is "trust" (item 2). The probability of trust in a partner increases with the expectation of benefits from trust-based action. Putnam [55] speaks of the standard of generalized reciprocity; these standards ensure that hospital employees work together by causing them to behave cooperatively [54]. Item 4 looks at perceptions of the quality of the atmosphere at work. Reciprocal behaviour (item 5) is a form of exchange, commonly referred to as a *quid pro quo *and forms the basis of what is called "a contingent relationship." In other words, workplace relationships lead to staff feeling obliged to the organization to act reciprocally. According to Coleman [70], the decisive prerequisite for solving problems using cooperation is the amount of social capital [71]. The common values within the hospital are emphasized in item 6.

#### Statistical methods

We performed stepwise multivariate linear regression using SPSS 15.0. If the proportion of missing values for a variable was < 25%, a mean value was imputed; variables with > 25% of missing values were excluded from analysis [72]. Variables with an intercorrelation > 0.8, which is an indicator for problems of collinearity, would also have been excluded from analysis, but no variable fulfilled this (both) criteria either [73]. The following analysis was conducted in a three step manner, as follows: first, we give an overview of descriptive values of all analysis variables, in the second step, we show Pearson product moment correlation coefficients (PMCC) for correlations between all variables to demonstrate bivariate relationships and to control for multicollinearity; and in the final analysis, we computed a multiple linear stepwise regression model on "job satisfaction."

## Results

163 respondents were male (58.8%) and 114 (41.2%) female. The average age was 40.0 years (standard deviation 9.9 years). 89 of the respondents (32.1%) had up to five years of professional experience, 111 (40.1%) had 6–16 years, and 77 (27.8%) had 17 years or more. 82 physicians (29.6%) specialize in internal medicine (e.g., cardiology and oncology), 57 physicians (20,6%) in visceral and vascular surgery, 40 physicians (14.4%) in neurology, psychiatry, and psychosomatics, 29 physicians (10.4%) specialize in other fields (e. g. gynaecology and paediatrics). 69 physicians (25.0%) hadn't specified their speciality.

Table 2 describes the constant independent analysis variables and the dependent variable, "job satisfaction," the distribution of which was approximately normal; the Kolmogorov-Smirnov test demonstrated no significant deviation from a normal distribution.

**Table 2:** Measurements of the variables "social capital," "workload," and "job satisfaction"

**Variable**	**Measurement**	**M**	**SD**	**Min**	**Max**
Social capital in hospital	Total score of "social capital in organizations" according to Pfaff et al. [68]	13.8	3.7	6	24
Workload	Total score of "intensity of labour" according to Richter et al. [69]	19.3	3.2	7	24
Job satisfaction	Single item according to Pfaff et al. [68]	4.74	1.3	1	7

M = Mean; SD = Standard deviation; Min = Minimum; Max = Maximum

In the second step of the analysis, we want to give an overview of the Pearson product moment correlation (PMCC) of all analysis variables (see table 3). This step followed two aims: 1) show bivariate relationships between all analysis variables, and 2) control for multicollinearity of variables used in the final regression model.

**Table 3:** Intercorrelation coefficients for all ordinal and metric analyses variables

	1. Age	2. Profess. experience	3. Workload	4. Social capital	5. Job satisfaction
1. Age		.736**	.073	.166**	.296**
2. Profess. experience			.055	.114	.285**
3. Workload				-.176**	-.277**
4. Social capital					.524**

** = Correlation is significant at the 0.01 level (2-tailed)

The intercorrelations ranged between .055 (workload with professional experience) and .736 (age with professional experience). Seven of 10 relationships were significant at the 0.01 level (2-tailed). The link between social capital and job satisfaction as a main topic of this analysis proved to be highly significant on a bivariate level (.524). All in all, no variable in the present study reached a critical correlation with another variable of > .8, which is discussed with regard to multicollinearity [73].

Regarding collinearity diagnostics, the magnitude of the intercorrelations among variables, is just one of the indicators for this problem. Therefore, additional indicators such as the tolerance of the variables and the variance inflation factor (VIF) are presented in table 4. The tolerance value shows the extent to which the independent variable in the corresponding line is predictable by other variables included in the model or whether it correlates with them. Tolerance values not lower than 0,1 or VIF-values up to 10 (VIF = reciprocal of tolerance value) [73] may be accepted. In this regression model no problems of collinearity arise, as all tolerance values are higher than 0,1 and all VIF-values are significantly lower than 10.

**Table 4:** Results from hierarchical multiple linear regression

		**Unstand**. **coefficients**		**Stand**. **coefficients**	**Collinearity statistics**		**Sign**. **level**	**Corr**. **R**^**2**^	**R^**2**^-changes**
		**B**	**Standard error**	**Beta**	**Tolerance**	**VIF**			
									
**1**	(Variable)	3.44	.408				.000***	**0.089**	**.099**
	gender	-.086	.151	-.033	.971	1.03	.569		
	**age**	.024	.011	.183	.451	2.21	.033*		
	professional experience	.242	.140	.146	.458	2.18	.086		
**2**	(Variable)	5.875	.582				.000***	**0.180**	**.091**
	gender	-.180	.144	-.069	.958	1.04	.214		
	**age**	.026	.010	.199	.451	2.21	.015*		
	professional experience	.243	.133	.147	.458	2.18	.069		
	**workload**	-.124	.022	-.308	.981	1.01	.000***		
**3**	(Variable)	3.392	.582				.000***	**0.366**	**.186**
	gender	-.128	.127	-.049	.956	1.04	.314		
	age	.015	.009	.116	.444	2.25	.108		
	professional experience	.255	.117	.155	.458	2.18	.030*		
	workload	-.089	.020	-.222	.944	1.05	.000***		
	**social capital**	.155	.017	.445	.935	1.07	.000***		

*Dependent variable: job satisfaction; * = p ≤ 0.05; ** = p ≤ 0.01; *** = p ≤ 0.001

The last analysis is computed with a 3-step hierarchical multiple linear regression model with overall job satisfaction as dependent and age, gender, professional experience, workload, and social capital as independent variables (see table 4). In the first step, only socio-demographic variables (age, gender, and professional experience) are introduced in the model. In the second step, workload is additionally introduced, and in the third step, social capital is added.

In the first step of the regression model, the explained variance of the dependent variable, job satisfaction, accounted for nearly 9%, and only age reached a significance level of p < .05. In the second step, the explained variance increased up to 18%. Besides workload, age remained significant. In the third step, the explained variance increased up to 37%. Significant information for explaining the dependent variable, job satisfaction, was given by the independent variables, professional experience, workload, and social capital. These variables explained > 1/3 of the variance of the dependent variable job satisfaction.

**Figure 1:**
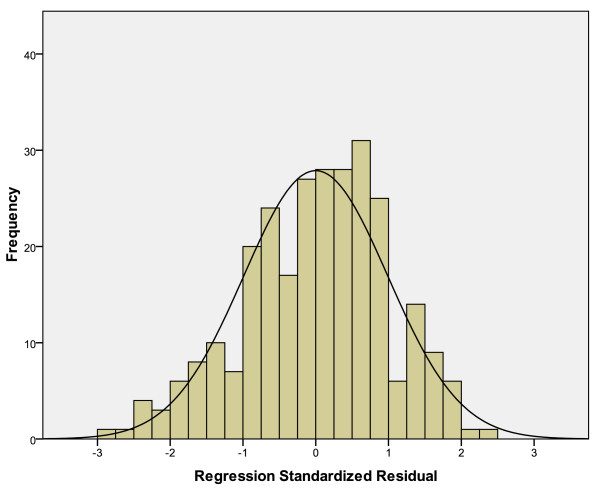
**Histogram on residuals with superimposed normal curve**.

In order for a multiple linear regression analysis to be appropriate, it is important to conduct a search focused on residuals to look for evidence that the necessary assumptions – i. e. normality and homogeneity of variance (homoscedasticity) – are not violated. Figure 1 shows the frequency of certain residuals. The value "0" indicates that no prediction error occurs. Negative values are corresponding to errors of overestimation, and positive values to errors of underestimation. If the residuals appear at random – as it is the case in this study -, the distribution of their frequency of occurence should converge to a normal distribution.

**Figure 2:**
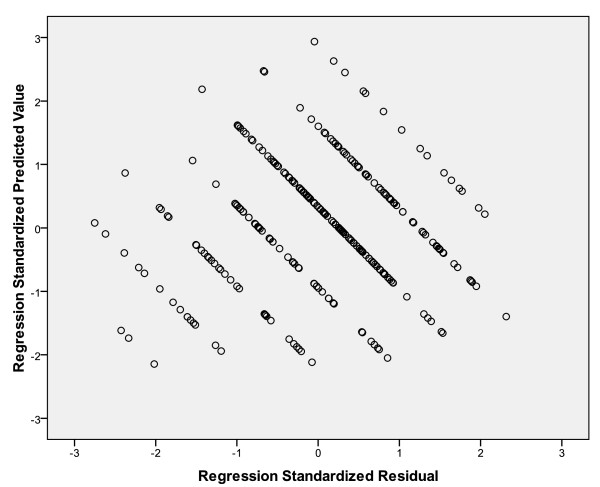
**Cumulative probability plot of the residuals; relationship between the predicted and residual values**.

Furthermore there should be no relationship between the predicted and residual values in the cumulative probability plot of the residuals. The residuals should be randomly distributed about the horizontal straight line through zero. Figure 2 shows the cumulative probability plot of the residuals and confirms the assumption of homoscedasticity.

## Discussion

To our knowledge, there is no existing literature to date that has explicitly examined the relationship between social capital in hospitals and physician job satisfaction. Therefore, this article extends prior research of social capital in the health care industry by examining the relationship between social capital at the workplace and job satisfaction of clinicians. Our analysis demonstrates that not only subjective workload and professional experience show a statistical significant correlation with job satisfaction, but also social capital in the hospital. Trust, mutual understanding, and shared aims are qualities of social capital, which unify members of social networks and communities and enable them to act cooperatively. Investment in the social capital of an organisation, e.g., a hospital, is a valuable investment in the social system, since the social capital, as shown in this analysis, has a significant impact on job satisfaction. On the basis of the recent literature, it is to be assumed that job satisfaction, in turn, affects well-being and health of an organisation's members and therefore the efficiency of the organisation itself. Furthermore, it becomes evident that job satisfaction is significantly associated with professional experience. The reason for this may be partly due to a "survival" function, which means that physicians who have found more strategies to maintain their satisfaction are more likely to survive a full career as a physician. It is argued that the higher satisfaction of physicians in later career stages results from "weeding out" the less satisfied physicians [15]. It has been further shown that subjective workload is associated with job satisfaction, such that the lower the workload, the higher the job satisfaction. Like many other studies, this analysis confirms again that clinicians consider their workload generally to be high (19.3 points on average of a maximum of 24). In contrast, sociodemographic variables, such as age and gender, did not have a significant impact.

### Limitations of the study

The current study had methodological limitations that may have affected interpretation of the results. Our cross-sectional design allowed identification of several factors associated with job satisfaction, although causal inferences can hardly be made. Since job satisfaction, as the dependent variable, and all predictor variables were assessed by self-reports, the results might be contaminated by common method variance or self-report bias [74]. Whether the results are applicable to other hospitals is difficult to assess. The selection of the four hospitals intentionally included hospitals in East and West Germany, and hospitals providing maximum and basic healthcare services in an attempt to achieve a form of guided random sampling of German hospitals and hospital-based physicians. On a positive note, a high response rate of 61% was achieved.

## Conclusion

Increasing social capital in hospitals requires in-house strategies for reinforcing a culture of trust and willingness to work together. Problems in the hospital or with individuals should be identified using regular, standardized employee polls. We propose regular "team sessions" and professional supervision as suitable measures for enhancing the social climate in hospitals and for improving communication structures.

In addition we assume, as often "the rot starts at the top," that the leaders in the middle and upper management levels in hospitals can contribute considerably to strengthen social capital. Management seminars should impart skills of optimising communication structures and processes in the hospital, detecting problems arising in the interaction between staff members and teamwork early, and reacting adequately. Thus, leaders can contribute effectively to improve the working atmosphere significantly in a top-down approach and thus act as role models for the next generation of leaders. This is an important, but too often disregarded task of physicians in leadership positions, especially in hospitals. Furthermore we suggest interventions implemented or designed not only on the individual level (i.e. physicians/leaders) but also on the organizational level. For example, hospital settings should be designed so that there is ample interaction and cooperation among health professionals, emphasizing trust, reciprocity, alliances, bonding, and shared understanding, while promoting organizational justice and conflict resolution. Additionally, we recommend assessing physical and mental symptoms (e.g., burnout, stress) and job satisfaction with standardized instruments within the scope of regular examinations by the hospital medical officer. In this way, first signs of weak social capital or a bad working atmosphere and the potential physical or mental effects on the staff members can be detected early.

## Competing interests

The authors declare that they have no competing interests.

## Authors' contributions

HP designed the study. OO performed the analysis, interpreted the results, and drafted the manuscript. ED interpreted the results, and drafted the manuscript. NE, PS, HP supervised the analysis, helped to interpret the results and participated in the formulation of the discussion. CK, TK, MN helped to interpret the results and contributed to the discussion. All authors reviewed and edited the manuscript for intellectual content.

## Pre-publication history

The pre-publication history for this paper can be accessed here:


